# Generation of infectious recombinant Adeno-associated virus in *Saccharomyces cerevisiae*

**DOI:** 10.1371/journal.pone.0173010

**Published:** 2017-03-29

**Authors:** Daniel Barajas, Juan Jose Aponte-Ubillus, Hassibullah Akeefe, Tomas Cinek, Joseph Peltier, Daniel Gold

**Affiliations:** 1 BioMarin Pharmaceutical Inc., Novato, California, United States; 2 Keck Graduate Institute, Claremont, California, United States; University of Nantes, FRANCE

## Abstract

The yeast *Saccharomyces cerevisiae* has been successfully employed to establish model systems for a number of viruses. Such model systems are powerful tools to study the virus biology and in particular for the identification and characterization of host factors playing a role in the viral infection cycle. Adeno-associated viruses (AAV) are heavily studied due to their use as gene delivery vectors. AAV relies on other helper viruses for successful replication and on host factors for several aspects of the viral life cycle. However the role of host and helper viral factors is only partially known. Production of recombinant AAV (rAAV) vectors for gene delivery applications depends on knowledge of AAV biology and the limited understanding of host and helper viral factors may be precluding efficient production, particularly in heterologous systems. Model systems in simpler eukaryotes like the yeast *S*. *cerevisiae* would be useful tools to identify and study the role of host factors in AAV biology. Here we show that expression of AAV2 viral proteins VP1, VP2, VP3, AAP, Rep78, Rep52 and an ITR-flanked DNA in yeast leads to capsid formation, DNA replication and encapsidation, resulting in formation of infectious particles. Many of the AAV characteristics observed in yeast resemble those in other systems, making it a suitable model system. Future findings in the yeast system could be translatable to other AAV host systems and aid in more efficient production of rAAV vectors.

## Introduction

Adeno-associated viruses (AAV) are single stranded DNA, non-enveloped, icosahedral viruses belonging to the genus *Dependoparvovirus*, family *Parvoviridae*. First discovered as contaminants of adenovirus preparations, they require co-infection with adenovirus or herpes virus for efficient replication and are asymptomatic in humans [1]. Several characteristics have made it possible to develop gene delivery vectors based on AAV and a number of gene therapy treatments for human diseases are being developed using recombinant AAV (rAAV) vectors [2–5]. AAV capsids are composed of 60 units of VP1, VP2 and VP3 in an approximate ratio of 1:1:10. The three proteins are produced from the cap gene through a combination of alternative splicing and leaky scanning of transcripts from the p40 promoter and they all share the C terminal sequence. An additional protein called assembly-activating protein (AAP) is translated from a different open reading frame in the cap gene and is required for capsid assembly [6]. Replication and encapsidation of the AAV DNA requires the inverted terminal repeats (ITRs) and the activity of Rep proteins. Four Rep proteins are produced from the Rep gene. The large ones, Rep78 and Rep68, are made from transcripts originating from the p5 promoter. The small ones, Rep52 and Rep40, are made from transcripts of the p19 promoter [3]. The long Rep proteins are essential for replication and can cleave the ITRs at the terminal resolution site (trs) and prompt isomerization of the ITRs for priming of the new DNA strand synthesis. The short Rep proteins are important for accumulation of the AAV DNA and are involved in the encapsidation of the AAV DNA [7,8]. Although the four Rep proteins show differences in enzymatic activity [7], only one large and one small Rep proteins seem to be sufficient for rAAV production [8–11]. AAV depends on functions provided by other viruses for efficient replication [12,13]. Adenovirus and herpes virus are the best understood helper viruses and co-infection with these viruses or co-expression of a subset of genes can provide the helper functions needed. The minimum set of genes from these viruses required to support AAV replication has been defined as E1A, E1B55K, E2A, E4orf6 and VA RNA for adenovirus [14,15] and UL5, UL8, UL29, UL30, UL42 and UL52 for herpes virus [16]. In the case of herpes virus the minimum set of genes seem to provide functions directly involved in replication including DNA polymerase, helicase and single stranded DNA (ssDNA) binding. In the case of adenovirus most helper genes seem to act indirectly by modifying the cellular environment, modulating AAV promoters or altering Rep activity and only E2A has an activity (ssDNA binding) that suggests a direct role in AAV replication [17]. Interestingly, rAAV vectors can be produced efficiently in baculovirus infected insect cells, suggesting that baculovirus vectors can provide the basic helper functions [11]. AAV likely relies also in multiple host factors at all stages of its life cycle. Understanding the roles of host factors is important for AAV biology and for rAAV vector production, especially in non-native host systems like baculovirus-insect cells where the lack of certain host factors could be limiting productivity. A number of host proteins associated with AAV have been identified using different approaches like co-immunoprecipitation and subcellular fractionation [18–21] however, for the majority of proteins, little is known about their role in the AAV life cycle. Protein complexes shown to be part of the AAV replication complex include DNA polymerase D, proliferating cell nuclear antigen (PCNA), replication factor C (RFC), the single-stranded DNA binding protein RPA and minichromosome maintenance complex (MCM) [18–20]. Another major group of factors interacting with AAV Rep proteins and DNA is the DNA damage response proteins including the MRN complex [22,23], however their significance in the AAV life cycle seems complex and is only partially understood [21,24].

The identification and characterization of host factors affecting eukaryotic viruses is a daunting task. The high complexity of higher eukaryote systems, cumbersome gene knockout methods and the functional redundancies that preclude appearance of a phenotype make the study of host factors in native virus-host systems very complex. The generation of model systems in simpler eukaryotes like the yeast *Saccharomyces cerevisiae* that recreate some aspects of the life cycle of higher eukaryote viruses has been a powerful tool to identify and characterize the role of host factors [25–27]. In many cases host factors identified in the model systems have been validated in the native host [28] confirming the utility of yeast as a model system platform. Yeast has been used also for production of virus-like particles (VLPs) of a number of viruses [29], illustrating the capability of yeast to fold proteins correctly and support multimerization and capsid assembly [30–32]. The utility of yeast as model system for AAV has been explored before [33,34]. Cervelli et al. [33], showed formation of ssDNA in yeast from a plasmid containing an ITR-flanked URA3 gene that was dependent on the presence of ITRs and expression of Rep proteins. Backovic et al. [34], showed formation of AAV capsids in yeast transformed with the AAV2 Cap gene plus a VP1 expression cassette driven by a GAL1 promoter. In the present work, six AAV2 proteins (VP1, VP2, VP3, AAP, Rep78 and Rep52) were expressed simultaneously in yeast together with a plasmid carrying a GFP gene flanked by ITRs. The results shown here demonstrate 1) formation of AAV2 capsids dependent on efficient expression of VP proteins and AAP; 2) rescue, replication and encapsidation of the ITR-flanked GFP DNA and 3) formation of infectious rAAV virions in yeast.

## Materials and methods

### Plasmids for expression of AAV proteins in *Saccharomyces cerevisiae*

The coding regions for AAV2 capsid and replication proteins were cloned into 2 micron pESC plasmids (Agilent Technologies) under the control of galactose-inducible promoters. The VP3 coding region was amplified by PCR from plasmid pAAV-RC2 (Cell Biolabs) with primers DB003 and DB005 (S2 Table). The resulting product was digested with *Bam*HI and *Sal*I and cloned into *Bam*HI and *Sal*I-digested pESC-HIS to generate DB021-pESC(H)-VP3 (S1 Table). The coding region for assembly-activating protein (AAP) was amplified by PCR with primers DB004 and DB007 or with primers DB004 and DB008, the products digested with *Spe*I and *Sac*I and cloned into DB021-pESC(H)-VP3 digested with *Spe*I and *Sac*I to generate plasmids DB025-pESC(H)-VP3-AAP and DB232-pESC(H)-VP3-AAP-HA respectively. A codon-optimized version of AAP was amplified with primers DB044 and DB045 or with primers DB044 and DB046. The products were digested with *Spe*I and *Sac*I and cloned into DB021-pESC(H)-VP3 digested with *SpeI* and *Sac*I to generate plasmids DB046-pESC(H)-VP3-AAP(op) and DB233-pESC(H)-VP3-AAP(op)-HA.

The Rep78 coding sequence was amplified by PCR from pAAV-RC2 (Cell Biolabs) with primers DB009 and DB011. The product was digested with *Apa*I and *Xho*I and ligated into pESC-TRP (Agilent Technologies) digested with *Apa*I and *Xho*I, generating plasmid DB023-pESC(T)-Rep78. A codon-optimized version of Rep78 was amplified with primers DB086 and DB088, the product digested with *Apa*I and *Xho*I and ligated into pESC-TRP to generate DB101-pESC(T)-Rep78(op). The VP2 coding sequence was amplified by PCR with primers DB002 and DB005. The product was digested with *Spe*I and *Bgl*II and ligated into DB023-pESC(T)-Rep78 or DB101-pESC(T)-Rep78(op) digested with *Spe*I and *Bgl*II to generate DB029-pESC(T)-Rep78-VP2 and DB135-pESC(T)-Rep78(op)-VP2 or ligated into pESC-TRP digested with *Spe*I and *Bgl*II to generate DB028-pESC(T)-VP2. A codon-optimized VP2 coding sequence was amplified with primers DB069 and DB073, digested with *Spe*I and *Bgl*II and ligated into DB023-pESC(T)_Rep78 to generate DB081-pESC(T)-Rep78-VP2(op).

The Rep52 coding sequence was amplified by PCR from pAAV-RC2 with primers DB010 and DB011. The product was digested with *Apa*I and *Xho*I and cloned into pESC-LEU (Agilent Technologies) digested with *Apa*I and *Xho*I, generating DB022-pESC(L)-Rep52. Alternatively, a codon-optimized version of Rep52 was amplified with primers DB087 and DB088, the product digested with *Apa*I and *Xho*I and ligated into pESC-LEU to generate DB102-pESC(L)-Rep52(op). The VP1 coding region was amplified by PCR from pAAV-RC2 with primers DB001 and DB005. The product was digested with *Spe*I and *Bgl*II and ligated into DB022-pESC(L)-Rep52, DB102-pESC(L)-Rep52(op) or into pESC-LEU digested with *Spe*I and *Bgl*II to generate DB027-pESC(L)-Rep52-VP1, DB228-pESC(L)-Rep52(op)-VP1 and DB026-pESC(L)-VP1 respectively. The intergenic region between *S*. *cerevisiae MRL1* (YPR079W) and *TEF1* (YPR080W) was amplified with primers DB113 and DB264 and digested with *Bam*HI and *Pac*I. The VP1 product digested with *Spe*I and *Bgl*II and the intergenic region product digested with *Bam*HI and *Pac*I were ligated into DB102-pESC(L)-Rep52(op) digested with *Spe*I and *Pac*I to generate DB138-pESC(L)-Rep52(op)-VP1-TEFp.

For cloning of the *Spodoptera frugiperda* immunophilin homolog FKBP46 [35], a cDNA was synthesized using ProtoScript II reverse transcriptase (New England Biolabs), primer DB286 and purified *S*. *frugiperda* RNA as template. The cDNA mixture was used as template for PCR with primers DB283 and DB284b and the product was digested with *Not*I. The intergenic region between *S*. *cerevisiae MRL1* (YPR079W) and *TEF1* (YPR080W) containing a polyadenylation signal and the TEF1 promoter was amplified by PCR using primers DB296 and DB299 using *S*. *cerevisiae* DNA as template. The resulting product was digested with *Not*I and ligated with the *Not*I-digested FKBP46 product. The ligation mixture was used as template for PCR with primers DB296 and DB300. The product was cloned by Gibson Assembly (New England Biolabs) into DB046-pESC(H)-VP3-AAP(op) that had been previously digested with *Sac*I and *Pac*I, resulting in plasmid DB155-pESC(H)-VP3-AAP(op)-TEF1p-FKBP46-HA.

The upstream region of *S*. *cerevisiae ADH2* (YMR303C) containing the ADH2 promoter (ADH2p) was amplified by PCR with primers DB149 and DB150. A DNA fragment from pESC-TRP1 containing the TRP1 polyadenylation signal was amplified by PCR with primers DB147 and DB148. The two products were fused by Gibson Assembly and used as template for PCR with primers DB303 and DB304. The product was digested with *Sac*I and *Bsp*EI. The human Adenovirus type 2 E2A coding region, codon-optimized for expression in *S*. *cerevisiae*, was amplified by PCR with primers DB305 and DB306 and then digested with *BspE*I and *Pac*I. The digested TRP1t-ADH2p and E2A fragments were ligated into DB138-pESC(L)-Rep52(op)-VP1-TEFp digested with *Sac*I and *Pac*I, generating plasmid DB149-pESC(L)-Rep52(op)-VP1-ADH2p-E2A(op)-HA. The upstream region of *S*. *cerevisiae GAL7* (YBR018C) was amplified using primers DB301 and DB302 and digested with *Sac*I and *Bsp*EI. The VP1 coding region was amplified from pAAV-RC with primers DB346 and DB347 and digested with *Bsp*EI and *Pac*I. The codon-optimized VP1 was amplified with primers DB376 and DB377 and digested with *Bsp*EI and *Pac*I. The E2A coding region was amplified with primers DB348 and DB349, digested with *Spe*I and *Sal*I and ligated into DB138-pESC(L)-Rep52(op)-VP1-TEFp digested with *SpeI*I and *Sal*I. The resulting plasmid was digested with *Sac*I and *Pac*I and ligated with the *Sac*I and *Bsp*EI-digested GAL7 product and the *Bsp*EI and *Pac*I-digested VP1 or VP1(op) products to generate DB205-pESC(L)-Rep52(op)-GAL10p-E2A-GAL7p-VP1 and DB220-pESC(L)-Rep52(op)-GAL10p-E2A-GAL7p-VP1(op) respectively.

To generate a plasmid containing the AAV DNA template, the *S*. *cerevisiae* 2 micron origin of replication and the URA3 gene were amplified by PCR from plasmid pESC-URA (Agilent Technologies) with primers DB035 and DB036. The product was digested with *Plu*TI and *Ngo*MIV and cloned into pAAV-GFP (Cell Biolabs) that had been digested with *Plu*TI and *Ngo*MIV, generating DB040-pAAV-GFP-2mic-URA3.

### Yeast cultivation and analysis of AAV protein and DNA products

Transformation of *S*. *cerevisiae* YPH501 (Matα/a, *ura3-52*, *lys2-801*^*amber*^, *ade2-101*^*ochre*^, *trp1-Δ63*, *his3-Δ200*, *leu2-Δ1*) with up to 4 plasmids was done following a PEG3350/Lithium Acetate procedure. Transformed colonies were selected in synthetic complete (SC) medium lacking histidine, leucine, tryptophan and uracil (HLTU-) and supplemented with 2% glucose. Transformed yeasts were pre-cultured for 24 hours in liquid SC (HLTU-) supplemented with 2% glucose, then cultured for 48 hours in liquid SC (HLTU-) supplemented with 2% galactose to induce expression of AAV proteins.

For analysis of protein accumulation 0.5 ml of culture was centrifuged and the yeast was resuspended in 0.1 M NaOH and incubated for 15 minutes at room temperature. Samples were centrifuged and resuspended in LDS loading buffer supplemented with DTT (Life Technologies) and incubated at 85°C for 5 minutes. Samples were run in NuPAGE 4–12% Bis-Acrilamide gels and transferred to PVDF membranes using an iBlot device (Life Technologies) following the manufacturer’s instructions. Membranes were incubated in blocking solution (Life Technologies) for 1 hour at room temperature, then in TTBS (25 mM Tris, 150 mM NaCl, 0.05% Tween 20) solution containing 1/1000 B1 anti-VP antibody (American Research Products), 1/300 303.9 anti-Rep antibody (American Research Products) or 1/5000 anti-HA antibody (GeneTex) for 1 hour and then in TTBS solution containing 1/5000 alkaline phosphatase (AP)-conjugated anti-mouse antibody for 1 hour. Membranes were washed in TTBS for 5 minutes between each antibody incubation and 4 times for 5 minutes after incubation with the secondary antibody. Membranes were incubated in BCIP/NBT solution for 5 to 30 minutes until protein bands were visible.

For analysis of VP protein accumulation in subcellular fractions transformed yeasts were pre-cultured for 24 hours in 12 ml HLTU- SC media with 2% glucose, then cultured for 40 hours in 150 ml HLTU- SC media with 2% galactose. Yeast cultures were collected by centrifugation and subcellular fractions were prepared as described [36]. Cytoplasmic and nuclear fractions were analyzed by SDS-PAGE and western blot as described above using B1 antibody for detection of VP proteins or anti-GAPDH antibody (GA1R, Pierce) for detection of GAPDH.

For analysis of DNA products total DNA was extracted from approximately 100 mg of yeast (4–5 ml of culture) using G-Biosciences yeast-geno-DNA kit. Nucleic acid samples were treated with RNase A to digest RNA and then run in Tris-Borate-EDTA (TBE) 0.8% agarose gels. Nucleic acids were transferred to nylon membranes using an iBlot device (Life technologies) following the manufacturer’s instructions. Nylon membranes were incubated in 0.4 M NaOH for 10 minutes to denature double-stranded DNA, then briefly washed in 1x SSC/0.1% SDS and crosslinked by UV irradiation (700 mJ). Membranes were then incubated in ULTRAhyb hybridization solution (Ambion) for 30 minutes at 65°C in rotating bottles and then in hybridization solution containing biotin-labeled DNA probes complementary to GFP sequence for 16 hours at 65°C. Biotin-labeled probes were generated using BrightStar Psoralen-Biotin kit (Ambion). Specific DNA products were detected using BrightStar Biodetect streptavidin-AP/CDP star kit (Ambion) following the manufacturer’s instructions.

### AAV purification and capsid quantification

Transformed yeasts were cultured as described above in a final volume of 200 ml. Cultures were centrifuged to collect yeast cells. Three grams of yeast were re-suspended in 4 ml of PBS supplemented with 0.25 M NaCl, 5 mM MgCl_2_ and yeast protease inhibitor cocktail (Yeast Protease-Arrest, G-Biosciences). Four grams of glass beads (425–600 μm, Sigma Aldrich) were added and the samples were shaken in a FastPrep device (MP Biomedicals) to break yeast cells. After that the samples were centrifuged at 100 g to remove unbroken cells. The resulting yeast lysate was supplemented with sarkosyl to a final concentration of 1%, triton X-100 to 0.5%, pluronic F68 to 0.05% and 200 units of benzonase, and incubated for 5 hours at 37°C to solubilize lipid membranes and digest unprotected nucleic acids. After that, the yeast lysate was mixed with 0.25 ml of AVB affinity resin (GE healthcare) and incubated for 30 minutes to allow binding of AAV capsids. The AVB resin was subsequently washed three times with a buffer containing Tris-HCl pH = 8.0, 0.5 M NaCl and 0.5% triton X-100. AAV capsids were eluted from the AVB resin first in 50 mM glycine pH = 2.5, 10% ethanol and 0.05% pluronic F68 and second in 20 mM Tris-HCl pH = 10.8, 0.5 M NaCl, 0.5 M arginine and 0.05% pluronic F68. Eluted fractions were pooled together and the pH adjusted to 7 by adding first or second elution buffers. Quantification of capsids in AVB eluates was done by ELISA following the manufacturer’s instructions (Progen) and using the manufacturer’s provided reference.

### Quantitative analysis of rAAV DNA

Digital droplet PCR (ddPCR) was used to quantify DNA in affinity purified and total DNA samples. Typically, samples were diluted 100 to 10000 to bring the target DNA concentration within ddPCR dynamic range. PCR reactions (25 μl) contained supermix for probes (BioRad), 0.5 mM of forward and reverse primers, 0.25 mM TaqMan probe and 1μl of the diluted sample. PCR reactions were processed in an automatic droplet generator (BioRad) before performing the PCR cycles. Droplets were analyzed in a QX200 droplet reader using QuantaSoft software package (BioRad). GFP DNA was quantified using primers and probe DB307, DB308 and DB309. URA3 was quantified using primers and probe DB406, DB407 and DB408. 18S rDNA was quantified using primers and probe DB438, DB439 and DB440. GFP and URA3 copy number in samples from total yeast DNA were reported relative to the 18S rDNA copy number in each sample for normalization.

### Analysis of AAV capsids, encapsidated DNA and infectivity

AAV capsid formation was analyzed by non-denaturing dot-blot using a capsid-specific antibody. Yeast lysates were prepared using glass beads as described above, serially diluted and 1 μl of each serial dilution applied onto nitrocellulose membranes. After letting sample spots dry, membranes were dumped in water and then blocked for one hour in blocking solution (Life technologies). Membranes were incubated in primary antibody solution (A20 anti-capsid 1/1000, American Research Products) for 1 hour at room temperature and then in secondary antibody solution (Alkaline phosphatase (AP)-conjugated anti-mouse 1/5000) for 1 hour. Membranes were washed in TTBS for 5 minutes between each antibody incubation and 4 times for 5 minutes after incubation with the secondary antibody. Membranes were incubated with CDP-star ready to use solution with nitro-block II (Applied Biosystems) and then imaged for chemiluminescence detection. Dot-blots for analysis of total VP proteins were performed in a similar way with the exceptions that yeast lysates were mixed with LDS-loading buffer and incubated at 85°C before spotting onto nitrocellulose and the primary antibody was B1 anti-VP (1/1000 dilution).

Cryoelectron microscopy of affinity purified samples was performed by NanoImaging Services (San Diego, CA). Briefly, three microliters of AVB affinity purified samples were applied to holey carbon films on 400-mesh copper grids and then processed in liquid ethane for sample vitrification. Electron microscopy of the vitrified samples was performed on a Tecnai T12 electron microscope at 120 kilo electron volts.

For the analysis of encapsidated DNA, affinity purified samples were supplemented with 5 mM MgCl_2_ and treated with benzonase to digest non-encapsidated nucleic acids. Benzonase was inactivated by adding EDTA to 10 mM and AAV capsids were lysed by adding SDS to 1% and incubating at 95°C for 5 minutes. Proteins were removed with phenol/chloroform and nucleic acids were ethanol precipitated in the presence of glycogen and resuspended in 1x loading buffer (New England Biolabs). For alkaline electrophoresis, samples were supplemented with NaOH to 30 mM, heated at 70°C for 10 minutes and run in 0.8% agarose gels in 30 mM NaOH, 20 mM EDTA. Afterwards gels were equilibrated on TBE buffer and the DNA transferred to nylon membranes using an iBlot device and DNA transfer stacks (Life Technologies). Membranes were crosslinked and incubated with biotin-labelled GFP probes as described above for specific detection of DNA.

For analysis of infectious rAAV virions in yeast samples HEK293 cells were cultured in DMEM media plus 10% serum to 70–90% confluency in 24 well plates. Cells were treated with 4 μM etoposide 1 day before addition of rAAV preparations. Affinity purified preparations from yeast were filter-sterilized using syringe-fitted 0.22 μm filters. AAV2-GFP produced in Sf9-baculovirus was obtained from Virovek. The titer of GFP DNA in all samples was determined by ddPCR as described above. HEK293 cultures were inoculated with 3 μl of each preparation after adjusting to 4E+10 GFP viral genomes (VG) per ml, or 3 μl of the control preparation that does not contain detectable GFP viral genomes. Four days after inoculation GFP-expressing cells were observed in a Carl Zeiss AxioVertA1 microscope. The percentage of cells expressing GFP was determined using an Accuri C6 flow cytometer (BD biosciences).

## Results

### Expression of AAV proteins in *Saccharomyces cerevisiae*

In natural hosts the AAV Rep proteins, VP proteins and AAP are expressed from the Rep and Cap genes at specific ratios through a combination of promoters, alternative splicing and leaky translation start at non-AUG codons. To bypass the need for efficient alternative splicing or leaky scanning in yeast, the coding regions for AAV2 Rep78, Rep52, VP1, VP2, VP3 and AAP, including a conventional AUG start codon, were cloned into 2micron plasmids under the control of galactose-inducible promoters so that each protein is expressed independently. Rep78, Rep52 and VP3 were cloned under the control of the GAL1 promoter, while VP1, VP2 and AAP were cloned under the control of the GAL10 promoter. To generate an AAV DNA template construct that can be transformed into yeast, a plasmid containing a CMVp-GFP cassette flanked by ITRs was modified to include the 2micron origin of replication and the URA3 auxotrophic marker. The four resulting plasmids can be co-transformed into yeast and are maintained at high copy number per cell.

**Fig 1:**
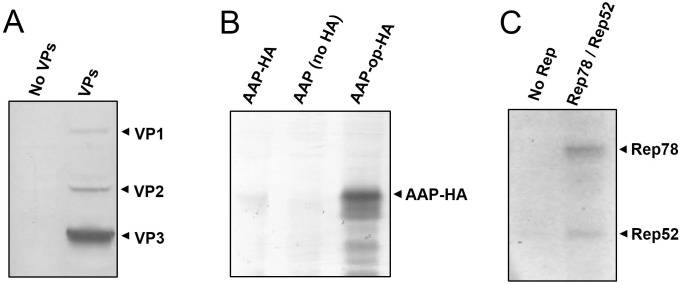
Detection of AAV proteins expressed in yeast. Yeast samples were processed for SDS-PAGE and western blot. (A) Detection of VP capsid proteins with B1 antibody. Samples from yeast carrying plasmids pESC-HIS, DB022, DB023 and DB040 (left lane, No VPs) or DB046, DB027, DB029 and DB040 (right lane, VPs). (B) Detection of HA-tagged AAP with anti-HA antibody. Samples from yeast carrying plasmids DB232, DB138, DB029 and DB040 (left lane, AAP-HA); DB046, DB228, DB029 and DB040 (middle lane, AAP no HA) or DB233, DB138, DB029 and DB040 (right lane, AAP-op-HA). (C) Detection of Rep proteins with 303.1 anti-Rep antibody. Samples from yeast carrying plasmids DB046, DB026, DB028 and DB040 (left lane, No Rep) or DB046, DB027, DB029 and DB040 (right lane, Rep78/Rep52).

To prevent potential negative impacts in yeast growth and protein expression caused by cytotoxic effects of AAV proteins, selection of yeast transformants and pre-growth were done under non-inducing conditions, using glucose as carbon source. Yeast cultures were then changed to media containing galactose as carbon source to induce expression of AAV proteins and 48 hours later protein expression was analyzed by western blot. Analysis with B1 anti-VP antibody reveled three major proteins, consistent with VP1, VP2 and VP3 (Fig 1A), with relative ratios of approximately 1:3:20. Detection of AAP was facilitated by expressing it as a fusion protein with a C-terminal HA tag and using anti-HA antibodies. Initial expression attempts using constructs with the native AAV2 AAP coding sequence showed very low or no detectable levels of AAP-HA (Fig 1B), even though the expression cassette included a conventional AUG start codon and a yeast-specific GAL10 promoter. However, AAP-HA was detectable when using a codon-optimized AAP coding sequence. The AAP coding region in the native AAV2 overlaps with that of VP2 and VP3. Selective pressure for the presence of optimal codons in VP2 and VP3 may be imposing the presence of suboptimal codons in AAP. Western blot analysis with 303.9 anti-Rep antibody showed two major proteins consistent with Rep78 and Rep52 expression (Fig 1C).

### AAV capsid formation

**Fig 2:**
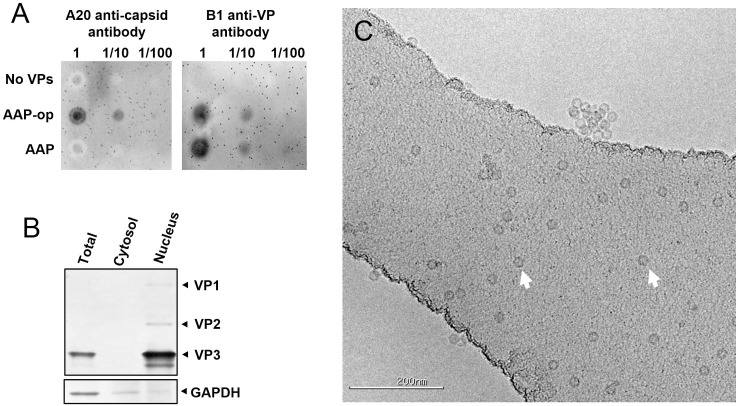
AAV capsid formation in yeast. (A) Yeast lysates (undiluted or diluted 1/10 and 1/100 fold) were spotted onto nitrocellulose membranes. Left panel: Detection of capsids with A20 capsid-specific antibody. Right panel: Detection of VP proteins with B1 antibody. Samples from yeast carrying plasmids pESC-HIS, DB022, DB023 and DB040 (No VPs); DB046, DB027, DB029 and DB040 (AAP-op) or DB025, DB027, DB029 and DB040 (AAP). (B) Western blot analysis of VP proteins in subcellular fractions from yeast carrying plasmids DB046, DB027, DB029 and DB040. VP proteins were detected using B1 antibody. GAPDH was detected using a specific antibody. (C) Electron microscopy of purified AAV capsids from yeast carrying plasmids DB046, DB027, DB029 and DB040. Examples of individual AAV capsids are pointed by arrows. Scale bar corresponds to 200 nm.

The importance of AAP for nuclear localization of VP proteins and capsid formation was shown before [6]. Previous studies by Backovic et al. [34] used *S*. *cerevisiae* to study AAV capsid formation. For that purpose yeast was transformed with the native AAV2 Rep-Cap expression cassette plus an additional cassette containing the VP1 coding region placed under a GAL1 promoter and with the native non-conventional start codon replaced to AUG. This work showed formation of detectable amounts of AAV capsids even without the addition of a dedicated AAP expression cassette. It is conceivable then that in that system enough AAP was produced from the native Rep-Cap cassette to facilitate some capsid formation. To investigate AAV capsid formation and the impact of AAP expression in our system, we analyzed capsid accumulation by dot-blot using A20 capsid-specific antibody in yeast transformed with constructs harboring the native AAP coding sequence or the codon-optimized one, plus cassettes for expression of the three VP proteins. Capsid accumulation was detectable in samples from yeast harboring the codon-optimized AAP cassette (Fig 2A, AAP-op). However no capsid accumulation was detectable above background levels in samples from yeast with the native AAP coding sequence (Fig 2A, AAP). Analysis of samples from the same yeast with B1 anti-VP antibody showed accumulation of similar amounts of VP proteins regardless of the AAP cassette used. This result supports that capsid formation in yeast is highly dependent on AAP. To study the subcellular localization of VP proteins, yeast transformed with constructs for expression of VP proteins and codon-optimized AAP was processed into sub-cellular fractions following a previously described method [36]. Nucleus enriched fraction as well as cytosol enriched fraction and non-fractionated yeast lysates were analyzed by western blot with B1 anti-VP antibody. VP proteins were clearly detectable in the nucleus enriched fraction but not in the cytosol enriched fraction, suggesting that VP proteins are efficiently transported to the nucleus (Fig 2B). Analysis by cryoelectron microscopy of affinity purified preparations from yeast showed abundant icosahedral particles of approximately 20 nm in diameter (Fig 2C), which were not present in preparations from yeast lacking VP proteins and AAP (not shown). All together the results confirm the formation of AAV capsids in yeast and the dependence on AAP for efficient capsid formation.

### AAV DNA rescue and replication

**Fig 3:**
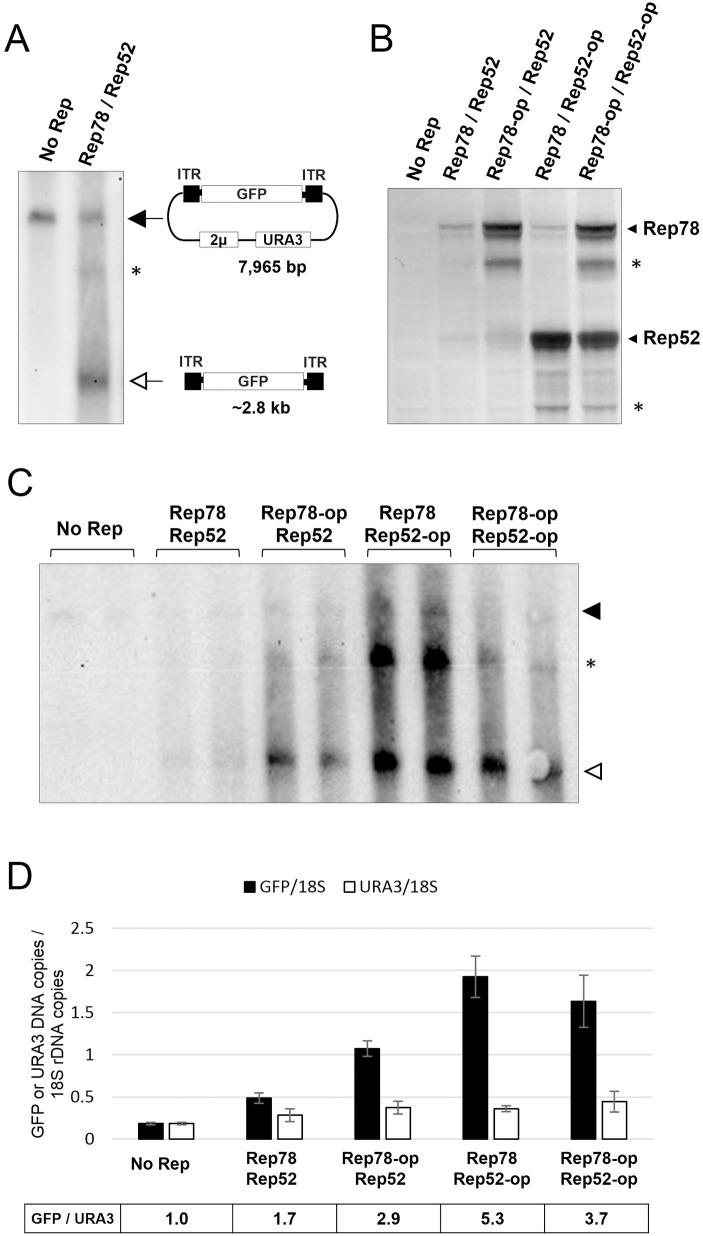
Replication of AAV DNA in yeast. (A) Southern blot with a GFP specific probe of yeast samples carrying plasmids DB046, DB026, DB028 and DB040 (No Rep) or DB046, DB027, DB029 and DB040 (Rep78, Rep52). The top band (black arrow) corresponds to plasmid DB040-pAAV-GFP-2mic-URA3 (pAAV-GFP/URA3). The bottom band (white arrow) likely corresponds to the rescued ITR-GFP-ITR DNA. The middle band (*) could be a dimer of the ITR-GFP-ITR DNA. (B) Western blot with 303.9 anti-Rep antibody of samples from yeast carrying plasmids DB046, DB026, DB028 and DB040 (No Rep); DB046, DB027, DB029 and DB040 (Rep78, Rep52); DB046, DB027, DB135 and DB040 (Rep78-op, Rep52); DB046, DB138, DB029 and DB040 (Rep78, Rep52-op); DB046, DB138, DB135 and DB040 (Rep78-op, Rep52-op). Additional bands not consistent with the expected size for Rep78 and Rep52 were also observed (*). (C) Southern blot with a GFP specific probe of yeast samples described above. See band descriptions in panel A and sample descriptions in panel B. (D) quantification by ddPCR of GFP and URA3 DNA species. ddPCR was performed with primers and TaqMan probes specific for GFP, URA3 and 18S ribosomal DNA sequences. Black bars indicate the average (2 to 3 samples) GFP copy number relative to 18S rDNA copy number. White bars indicate the average URA3 copy number relative to 18S rDNA. The ratio of GFP to URA3 copies is indicated below the diagram. See sample descriptions in panel B.

Previous studies by Cervelli et al. [33] showed that *S*. *cerevisiae* was capable to carry out single-stranded DNA synthesis from an ITR-containing template when Rep68 was expressed. Although the dependence on Rep68 and ITR presence suggested some resemblance to AAV replication, the observed DNA synthesis was not deemed as canonical AAV replication. We used southern blot to analyze in our system the potential rescue and replication from a plasmid of a GFP reporter gene flanked by ITRs. Samples from yeast containing the pAAV-GFP/URA3 plasmid showed a major band in southern blots consistent with the expected size of the plasmid (7,965 bp, Fig 3A). Samples from yeast expressing Rep78 and Rep52 proteins showed other smaller bands in addition to the one attributable to the pAAV-GFP/URA3 plasmid. The most abundant band had a size consistent with that expected for the rescued ITR-GFP-ITR DNA product (~2.8 kb). A larger, less abundant band, had approximately twice the size expected for the ITR-GFP-ITR DNA, suggesting it could be a dimer of the rescued ITR-GFP-ITR product. The intensity of the band is influenced by several factors in addition to the abundance of each DNA species, making quantification based on southern blot inaccurate. To quantify the amount of rescued ITR-GFP-ITR DNA in comparison to the pAAV-GFP/URA3 plasmid, we used digital-droplet PCR (ddPCR). We used primers for GFP to quantify the ITR-GFP-ITR rescued DNA plus the pAAV-GFP/URA3 plasmid, and primers for URA3 to quantify the pAAV-GFP/URA3 plasmid. The endogenous *ura3-52* gene is also recognized by URA3 primers but its contribution to the total number of URA3 copies per cell is negligible. Samples from yeast containing the pAAV-GFP/URA3 plasmid but no Rep proteins showed similar titers for GFP and for URA3, indicating there are no other GFP DNA copies in addition to the pAAV-GFP/URA3 plasmid (Fig 3D). However, samples from yeast expressing Rep78 and Rep52 showed in average 1.7 GFP DNA copies for each URA3 DNA copy, suggesting the presence of approximately 0.7 copies of rescued ITR-GFP-ITR for each copy of the pAAV-GFP/URA3 plasmid. In addition to expression of Rep78 and Rep52 driven by the natural coding sequences, we tried Rep protein expression from codon-optimized sequences. Analysis by western blot showed higher accumulation of Rep78 and Rep52 proteins in yeast when the codon-optimized sequences were used (Fig 3B). The number of ITR-GFP-ITR copies in comparison to pAAV-GFP/URA3 plasmid copies was impacted by the abundance of Rep proteins. Samples from yeast expressing Rep78 or Rep52 from codon-optimized sequences showed an average of 2.9 and 5.3 copies of GFP DNA for each copy of URA3 DNA respectively, suggesting the presence of 1.9 and 4.3 copies of ITR-GFP-ITR for each copy of the pAAV-GFP/URA3 plasmid (Fig 3C and 3D). Expression of both Rep78 and Rep52 from codon-optimized sequences did not further improve the accumulation of rescued ITR-GFP-ITR, with an average of 3.7 GFP DNA copies for each URA3 DNA copy or 2.7 ITR-GFP-ITR copies for each pAAV-GFP/URA3 plasmid. In addition to Rep proteins, efficient AAV DNA replication in animal cells relies on functions provided by helper viruses, as well as functions provided by certain host proteins. We tested the effect on AAV replication of adenovirus E2A protein and host immunophilin. Adenovirus E2A is a single-stranded DNA binding protein that could have a direct role in AAV DNA replication [17]. The human immunophilin FKBP52 was shown to bind the D sequence of the ITR and could exert an influence on AAV DNA replication and encapsidation [37, 38]. E2A and either FKBP52 or the armyworm *Spodoptera frugiperda* homolog FKBP46 were co-expressed in yeast with AAV proteins and pAAV-GFP/URA3 plasmid. Although some experiments showed increased accumulation of the rescued ITR-GFP-ITR product, a consistently reproducible effect was not observed (not shown). Nevertheless, expression of E2A and immunophilin was included in subsequent experiments.

### Encapsidation of rAAV DNA

**Fig 4:**
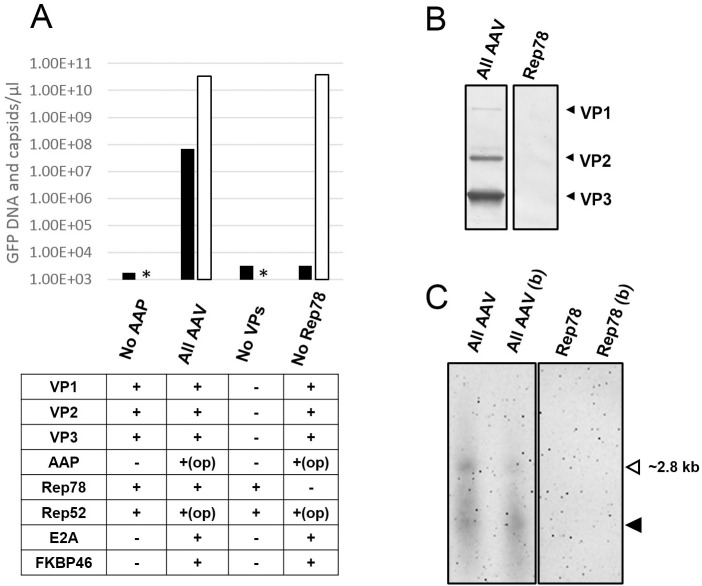
Encapsidation of AAV DNA. (A) Analysis of GFP DNA co-purified with AAV capsids. AAV capsids were purified by AVB affinity chromatography from yeast carrying plasmids DB021, DB027, DB029 and DB040 (No AAP); DB155, DB149, DB029 and DB040 (All AAV); pESC-HIS, DB022, DB023, DB040 (No VPs); DB155, DB149, DB028 and DB040 (No Rep78). GFP DNA was quantified by ddPCR while capsids were quantified by ELISA. Asterisks (*) indicate capsid content bellow the limit of detection. (B) Detection by western blot of VP proteins in affinity purified capsids from yeast carrying plasmids DB155, DB149, DB029 and DB040 (All AAV) or from yeast carrying plasmids pESC-HIS, pESC-LEU, DB081 and DB040 (Rep78). Note that plasmid DB081 contains a VP2 expression cassette, however VP2 alone does not form capsids and is not purified by AVB affinity chromatography. (C) Detection by southern blot of GFP DNA co-purified with AAV capsids. Samples were treated with benzonase (b) or not treated before DNA extraction and run as single stranded DNA on alkaline gels. A band of the expected size for the ITR-GFP-ITR rAAV DNA product was observed (white arrow) as well as additional material of smaller size (black arrow) which is likely the result of partial encapsidation [8]. Refer to panel B for sample descriptions.

DNA encapsidation is the process in which single stranded AAV DNA gets pumped into preformed capsids, and is regarded as a limiting rate step in the formation of AAV virions [8]. The data presented above shows that AAV capsids accumulate in the nucleus and rAAV DNA can be rescued from the plasmid pAAV-GFP/URA3 and possibly replicated. Therefore we next analyzed if some of the rAAV DNA is present inside AAV capsids. AAV capsids were purified from yeast lysates by affinity chromatography. The amount of GFP DNA co-purified was analyzed by ddPCR. GFP DNA was detectable in the affinity purified material from yeast expressing all AAV components. The amount of GFP DNA detected was 6.8E+7 copies/μl, while the amount of capsids determined by ELISA was 3.4E+10 capsids/μl, indicating a capsid to GFP DNA ratio of approximately 500. ddPCR results showed only background levels of GFP DNA in affinity purified material from yeast lacking either AAP, the three VP proteins or Rep78 (Fig 4A), suggesting that GFP DNA was only present if co-purified with AAV capsids. DNA was extracted from purified capsids, run on alkaline denaturing agarose gels and analyzed by southern blot with a GFP specific probe. DNA material of different sizes was detectable. The top band had a size consistent with that expected for the full-length ITR-GFP-ITR rAAV DNA product (~2.8 kb, Fig 4C), while the smaller DNA material could be the result of inefficient/partial encapsidation as observed in other systems [8]. In contrast, no DNA material was detectable in samples purified in the same manner from yeast expressing Rep78 but no capsids (Fig 4C, Rep78 lanes), suggesting the detected DNA is co-purified with the capsids. A large amount of the DNA material was resistant to nuclease degradation as observed in purified AAV capsids that were treated with benzonase before DNA extraction, supporting that a large fraction of the DNA co-purified with the AAV capsids is protected and therefore likely encapsidated.

### Infectivity of rAAV virions

**Fig 5:**
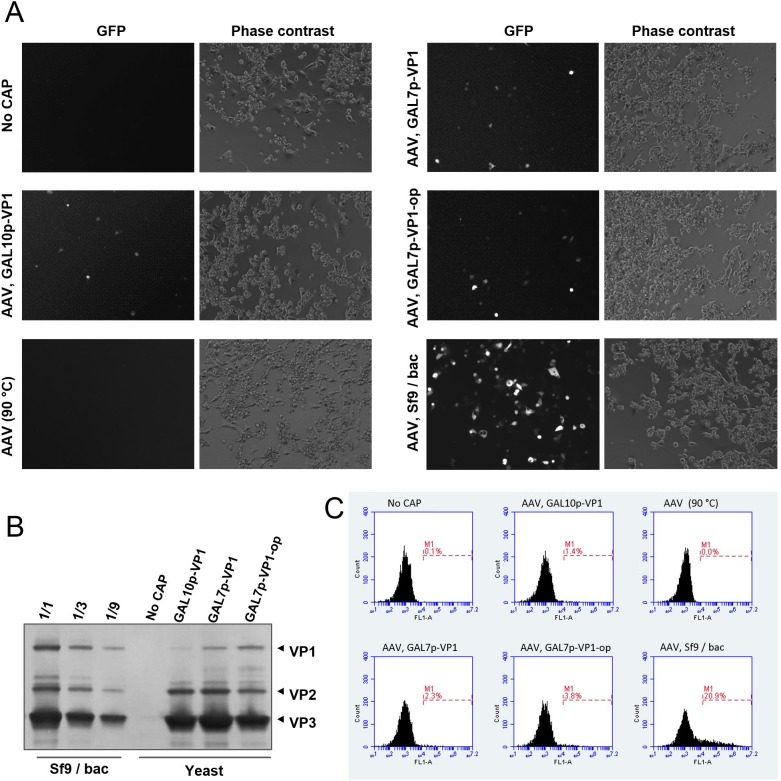
Infective rAAV virions in affinity purified fractions from yeast. (A) Detection of infectious rAAV virions in affinity purified fractions from yeast carrying plasmids DB155, DB149, DB029 and DB040 (AAV, GAL10p-VP1); pESC-HIS, DB102, DB023 and DB040 (No CAP); DB155, DB205, DB029 and DB040 (AAV, GAL7p-VP1); DB155, DB220, DB029 and DB040 (AAV, GAL7p-VP1-op). Affinity purified fraction treated at 90°C to denature capsids prior to addition to HEK293 cultures (AAV, 90°C). rAAV2-GFP produced in Sf9/baculovirus was used as a reference (AAV, Sf9/bac). HEK293 cells were treated with etoposide and transduced with 1.2E+8 viral genomes from each of the preparations. GFP expressing cells were observed 4 days after on a UV microscope. (B) Analysis with B1 anti-VP antibody of VP protein content in affinity purified fractions. rAAV produced in Sf9/baculovirus (Sf9/bac) was loaded in three fold serial dilutions for comparison. (C) Flow cytometry histograms showing percentage of cells expressing GFP.

The recombinant AAV DNA construct used to produce virions in yeast contains a GFP gene under the control of a CMV promoter which is active in mammalian cells. Therefore, the presence of infective rAAV virions can be tested by adding affinity purified fractions from yeast to HEK293 cell cultures and observing GFP expression after several days. GFP expression in some HEK293 cells was observed under UV microscope 4 days after addition of affinity purified fractions from yeast expressing all AAV components (Fig 5A). In contrast, no GFP expression was observed in HEK293 cultures inoculated with affinity purified fractions from yeast lacking capsid proteins. In addition, when the affinity purified fractions from yeast expressing all AAV components were treated at 90°C to denature AAV capsids before addition to HEK293 cultures no GFP expression was observed, suggesting that GFP expression in HEK293 cells is the result of transduction by rAAV virions and not transfection of naked GFP DNA. Analysis by flow cytometry indicated that 1.4% of cells expressed GFP as a result of rAAV transduction. For comparison, transduction with rAAV2-GFP produced in Sf9/baculovirus system resulted in 20.9% of cells expressing GFP (Fig 5C). VP1 contains a phospholipase activity that is essential for infectivity [39] and the amount of VP1 present in capsids has been shown to influence transduction efficiency [11]. To investigate if the amount of VP1 in yeast preparations is a factor in the observed transduction efficiency, we generated additional plasmid constructs for expression of higher amounts of VP1 using the stronger GAL7 promoter and codon optimization of the VP1 coding sequence. Western blot analysis of VP proteins in affinity purified rAAV confirmed that the modifications lead to a higher content of VP1 in AAV capsids (Fig 5B), although it was still lower than in rAAV produced in Sf9/baculovirus. Transduction of HEK293 cells with affinity purified rAAV from yeast harboring VP1 under GAL7 promoter or a codon-optimized VP1 under GAL7 promoter resulted in 2.3% and 3.8% of cells expressing GFP respectively (Fig 5C), supporting that the amount of VP1 in capsids is a factor affecting transduction efficiency. All together the results confirmed the presence of infectious rAAV virions in affinity purified fractions from yeast.

## Discussion

Adeno-associated viruses (AAV) are the focus of enormous interest because of their use as gene delivery vectors. Gene therapy applications will require production of large amounts of rAAV vectors with consistent quality. The use of model systems in simpler eukaryotes like yeast could lead to better understanding of rAAV vector biology, particularly the role of host factors, and that could be translated into major improvements in rAAV production regarding yield, purity and potency of vectors [40]. The yeast *S*. *cerevisiae* is an excellent model system: it has a small and well characterized genome containing mostly protein coding sequences, it is easy to manipulate genetically and easy to grow. A number of genome-wide libraries available and the lower functional redundancy of gene products compared to higher eukaryotes facilitate the identification of host factors. In line with it, the contribution of yeast model systems to the understanding of viruses like TBSV and BMV has been crucial [27,28,41–43].

The results reported here establish yeast as a model platform for studying AAV biology and rAAV vector production. Our system recapitulates major aspects of the AAV life cycle observed in other systems: 1) capsids are formed in the nucleus and require efficient expression of AAP; 2) A DNA flanked by ITRs can be rescued form a plasmid and replicated; 3) Some of the ITR-flanked DNA can be encapsidated forming rAAV virions capable of transducing HEK293 cells. Formation of AAV capsids in yeast has been reported before [34] using yeast transformed with the natural AAV2 cap gene plus an additional cassette for VP1 expression driven by a galactose inducible promoter. Although VP3 expression from the natural p40 promoter was detectable in yeast, it was likely suboptimal since VP1 expression from GAL1 promoter had to be induced only partially to achieve close to optimal VP1 to VP3 ratio. In our system we opted for bypassing any leaky scanning or alternative splicing requirements of the natural AAV2 cap gene by expressing each of the three capsid proteins independently using strong, inducible yeast promoters and conventional AUG start codons. The results showed expression of VP1, VP2 and VP3 at ratios close to the optimal 1:1:10. Our results also showed that efficient expression of AAP is essential for capsid assembly. Since the system described by Backovic et al. [34] showed formation of capsids, it is conceivable that some AAP was expressed in yeast from the AAV2 cap gene by translation initiation at the non-conventional CTG start codon [6]. Expression of VP2, which is also dependent on translation initiation at a non-conventional ACG codon, was detectable after capsid purification [34]. Single-stranded DNA synthesis dependent on ITR and Rep has also been observed in yeast before, however the characteristics of the newly synthesized DNA matched only partially those of rAAV DNA made in other systems [33]. Our system used a high copy number plasmid as a source of rAAV DNA and expression *in trans* of Rep proteins from yeast inducible promoters. The use of galactose inducible promoters prevented expression of Rep proteins during yeast transformant selection and pre-growth avoiding potential cytotoxicity problems during those stages. Using this configuration we observed low molecular weight DNA products matching the expected size for a double stranded monomer (~2.8 kb) indicating there is rescue of the rAAV DNA from the plasmid, and a double stranded dimer (~5.5 kb) [10] strongly suggesting replication of the rescued rAAV DNA. Interestingly the increase in Rep78 and Rep52 expression resulting from optimization of the coding sequence lead to a higher accumulation of the rAAV DNA, represented by the GFP sequence, without affecting the accumulation of the plasmid backbone, represented by URA3 sequence, further suggesting replication of the rAAV DNA. An alternative possibility is that the rescued rAAV DNA is selectively stabilized by the Rep proteins after being rescued from the pAAV-GFP/2mic-URA3 plasmid. However, to cause the observed results, in the presence of Rep proteins the pAAV-GFP/2mic-URA3 plasmid would have to be replicated to much higher copy numbers than observed in the absence of Rep proteins, then after ITR cleavage by Rep proteins the plasmid backbone would have to be selectively degraded and the rAAV selectively stabilized. A number of factors supported that some of the rAAV DNA gets encapsidated and rAAV virions are formed: 1) The rAAV DNA co-purified with affinity purified capsids is resistant to nuclease degradation, strongly suggesting it is inside the capsids; 2) Affinity purified preparations added to HEK293 cells resulted in transduction of GFP DNA dependent on the presence of intact AAV capsids. 3) The amount of GFP-expressing cells was impacted by the VP1 content of rAAV capsids, further supporting that the expression of GFP is the result of rAAV transduction. Encapsidation of rAAV DNA in yeast seems to happen with low efficiency, since the number of total capsids in affinity purified preparations was approximately 500 fold higher than the amount of co-purified rAAV DNA, suggesting that the cell environment and host factors in yeast or the amount or timing of Rep proteins expression is not optimal for efficient encapsidation. The infectivity of rAAV-GFP preparations made in yeast was lower in comparison to rAAV-GFP made in Sf9/baculovirus. In addition to the lower VP1 content of AAV2 capsids made in yeast, other possible reasons for the lower infectivity are the large excess of empty capsids, which might be competing for cell entry, or a higher prevalence of partially encapsidated DNA products, which would be quantified as viral genomes but would not result in GFP expression in transduced cells.

To the best of our knowledge at least one other laboratory has explored the use of yeast as a model system for AAV [44], in addition to us and the Galli laboratory [33,34]. Our system is to date the most complete regarding characterization and recapitulation of AAV key features. Our system can be readily used to study host factors affecting capsid formation, AAV replication and encapsidation. The use of yeast as a competitive platform for rAAV production is still far down the road and would require major improvements particularly in replication and encapsidation of the rAAV DNA.

## Supporting information

S1 Table Plasmids generated in this study. (DOCX)

S2 Table Primers used in this study. (DOCX)
