# Supplementary material for: Generation of infectious recombinant Adeno-associated virus in Saccharomyces cerevisiae
Source: PLoS One. 2017 Mar 29;12(3):e0173010. doi: 10.1371/journal.pone.0173010 (PMC5371294; doi:10.1371/journal.pone.0173010)
Supplement: S1 Table — (DOCX) [file pone.0173010.s001.docx]

**S1 Table. Plasmids generated in this study.**

| **Plasmid** | **expression cassettes** | **selection marker** |
| --- | --- | --- |
| DB021-pESC(H)_VP3 | GAl1p-VP3 | HIS3 |
| DB022-pESC(L)_Rep52 | GAL1p-Rep52 | LEU2 |
| DB023-pESC(T)_Rep78 | GAL1p-Rep78 | TRP1 |
| DB025-pESC(H)_VP3_AAP | GAl1p-VP3; GAL10p-AAP | HIS3 |
| DB026-pESC(L)_VP1 | GAL10p-VP1 | LEU2 |
| DB027-pESC(L)_Rep52_VP1 | GAL1p-Rep52; GAL10p-VP1 | LEU2 |
| DB028-pESC(T)_VP2 | GAL10p-VP2 | TRP1 |
| DB029-pESC(T)_Rep78_VP2 | GAL1p-Rep78; GAL10p-VP2 | TRP1 |
| DB040-pAAV-GFP_2mic-URA3 | CMVp-GFP | URA3 |
| DB046-pESC(H)_VP3_AAP(op) | GAl1p-VP3; GAL10p-AAP(op) | HIS3 |
| DB081-pESC(T)_Rep78_VP2(op) | GAL1p-Rep78; GAL10p-VP2(op) | TRP1 |
| DB101-pESC(T)_Rep78(op) | GAL1p-Rep78(op) | TRP1 |
| DB102-pESC(L)_Rep52(op) | GAL1p-Rep52(op) | LEU2 |
| DB135-pESC(T)_Rep78(op)_VP2 | GAL1p-Rep78(op); GAL10p-VP2 | TRP1 |
| DB138-pESC(L)_Rep52(op)_VP1_TEFp | GAL1p-Rep52(op); GAL10p-VP1 | LEU2 |
| DB149-pESC(L)_Rep52(op)_VP1_ADH2p-E2A(op)-HA | GAL1p-Rep52(op); GAL10p-VP1; AHD2p-E2A(op)-HA | LEU2 |
| DB155-pESC(H)_VP3_AAP(op)_TEF1p-FKBP46-HA | GAL1p-VP3; GAL10p-AAP(op); TEF1p-FKBP46-HA | HIS3 |
| DB205-pESC(L)_Rep52(op)_Gal10-E2A(op)_Gal7-VP1 | GAL1p-Rep52(op); GAL10p-E2A(op); GAL7p-VP1 | LEU2 |
| DB220-pESC(L)_Rep52(op)_Gal10-E2A(op)_Gal7-VP1(op) | GAL1p-Rep52(op); GAL10p-E2A(op); GAL7p-VP1(op) | LEU2 |
| DB228-pESC(L)_Rep52(op)_VP1 | GAL1p-Rep52(op); GAL10p-VP1 | LEU2 |
| DB232-pESC(H)_VP3_AAP-HA | GAL1p-VP3; GAL10p-AAP-HA | HIS3 |
| DB233-pESC(H)_VP3_AAP(op)-HA | GAL1p-VP3; GAL10p-AAP(op)-HA | HIS3 |

All plasmids contained a 2µ origin of replication for high copy maintenance in yeast and one of 4 auxotrophic markers (HIS3, LEU2, TRP1 or URA3), allowing for co-transformation of 4 plasmids. (p): promoter. (op): codon-optimized sequence. (HA): haemagglutinin tag.
