# Supplementary material for: Generation of infectious recombinant Adeno-associated virus in Saccharomyces cerevisiae
Source: PLoS One. 2017 Mar 29;12(3):e0173010. doi: 10.1371/journal.pone.0173010 (PMC5371294; doi:10.1371/journal.pone.0173010)
Supplement: S2 Table — (DOCX) [file pone.0173010.s002.docx]

**S2 Table. Primers used in this study.**

| **Primer name** | **Primer sequence** |
| --- | --- |
| DB001/VP1-AAV/SpeBam/F | GCCCactagtGGATCCaccATGGCTGCCGATGGTTATCTTC |
| DB002/VP2-AAV/SpeBam/F | GCCCactagtGGATCCaccAtGGCTCCGGGAAAAAAGAGGC |
| DB003/VP3-AAV/SpeBam/F | GCCCactagtGGATCCaccATGGCTACAGGCAGTGGC |
| DB004/AAP-AAV/SpeBam/F | GCCCactagtGGATCCaccatgggaCTGGAGACGCAGACTCAG |
| DB005/VP-AAV/SalSacBgl/R | cgccagatctGAGCTCgtcgacTTACAGATTACGAGTCAGGTATCTGG |
| DB007/AAP-AAV/SalSacBgl/R | cgccagatctGAGCTCgtcgacTCAGGGTGAGGTATCCATACTGTG |
| DB008/AAP-AAV/HA/SalSacBgl/R | cgccagatctGAGCTCgtcgacTTACGCGTAATCTGGAACATCGTATGGGTAgccGG  GTGAGGTATCCATACTGTG |
| DB009/Rep78-AAV/SpeApa/F | CGGCactagtGGGCCCaccATGCCGGGGTTTTACGAG |
| DB010/Rep58-AAV/SpeApa/F | CGGCactagtGGGCCCaccATGGAGCTGGTCGGGTG |
| DB011/Rep78-AAV/XhoBgl/R | cggcAGATCTctcgagTTATTGTTCAAAGATGCAGTCATCCAAATCC |
| DB035-URAt-NgoMIV-PluTI-R | cgcGGCGCCgccggcTTTCCTGATGCGGTATTTTCTCC |
| DB036-2mic-NgoMIV-BstAPI-PluTI-F | cgcGCCGGCgcaccatatgcGGCGCCACCTGAACGAAGCATCTGTG |
| DB044-AAPopt-SpeI-F | GCCCactagtaccATGGGCTTAGAAACGCAAAC |
| DB045-AAPopt-SacI-R | cgccGAGCTCTCACGGAGATGTATCCATGGAATG |
| DB046-AAPHAopt-SacI-R | cgccGAGCTCTTACGCGTAATCTGGAACATCGTATGGGTAgccCGGAGATGTATCC  ATGGAATG |
| DB069/VP2/SpeI/F | cggactagtCCGAAACGATGGCGCCTACCGGTAAACG |
| DB073/VP1/2/BglII/R | gccagatctCTATAATGGTCTAGTGAGATACCTAGTTC |
| DB086/Rep78(opt)/SpeApa/F | CGGCactagtGGGCCCGAAACGATGCCAGGTTTCTACGAAATAG |
| DB087/Rep52(opt)/SpeApa/F | CGGCactagtGGGCCCGAAACGATGGAATTAGTGGGATGGCTTG |
| DB088/Rep78(op)/XhoBgl/R | cggcAGATCTctcgagTTATTGTTCGAAGATACAATCATCCAAATCG |
| DB113/TEF1up/SacI/BamHI/F | CGGCgagctcggatccGGCTGATAATAGCGTATAAACAATGC |
| DB264/TEF1/BspEI/XmaI/PacI/R | cgcgTTAATTAAcgccccgggTCCGGATTTGTAATTAAAACTTAGATTAGATTGC |
| DB147/2mic/GB-help/F | GGTACATCTACGGATTACGAACGAAGCATCTGTGCTTCATTTTG |
| DB148/TRP1/GB-ADH2p/R | GGTGATTAGCCTGATGCGGTATTTTCTCCTTACG |
| DB303/TRP1t/BglII/SacI/F | gcgAGATCTgagctcCAAATTTCGTCAAAAATGCTAAGAAATAGG |
| DB304/ADH2p/BspEI/XmaI/R | gcgCCCGGGtccggaTGTGTATTACGATATAGTTAATAGTTGATAGTTG |
| DB305/E2A(op)/BspEI/F | CGCtccggaATGGCATCCAGAGAAGAGGAAC |
| DB306/E2op-HA//PacI/R | gcgcTTAATTAATTACGCGTAATCTGGAACATCGTATGGGTAGAAATCGAATGGGTT  CTGTCTGG |
| DB286/FKBP46/RT/R | catactggcacattcatcaacatttcac |
| DB283/FKBP46/NotI/F | ccGCGGCCGCatgttttggggacttattatggaaccgaac |
| DB284b/FKBP46/HA/PacI/R | cgcgTTAATTAAttaCGCGTAATCTGGAACATCGTATGGGTAtttcacattcttcaggt  caacttcaaatactag |
| DB296/MRL1t/GB-AAP2/F | TCCATGGATACATCTCCGTGAGAGCTCGGCTGATAATAGCGTATAAACAATGC |
| DB300/HA/GB-pESC/R | AAACCTCTGGCGAAGAATTGTTAATTAActaCGCGTAATCTGGAACATC |
| DB299/TEF1p/BspEI/NotI/R | ccGCGGCCGCTCCGGATTTGTAATTAAAACTTAGATTAGATTGCTATGCTTTCTTTC |
| DB149/ADH2p/GB-TRP1/F | GCATCAGGCTAATCACCATTCTAATGTTTTAATTAAGGGATTTTGTCT |
| DB150/ADH2p/GB-E1A/R | taatatgtctcatTGTGTATTACGATATAGTTAATAGTTGATAGTTGATTGTATGC |
| DB301/GAl7/BamHI/SacI/F | cgcGGATCCgagctcTTTTCCTGATTTGCCAGCTTACTATC |
| DB302/GAL7/BspEI/XmaI/PacI/R | CGCGttaattaaCCCGGGtccggaTTTTGAGGGAATATTCAACTGTTTTTTTTTATC |
| DB346/VP1-AAV/BspEI/F | GCCCTCCGGAaaaATGGCTGCCGATGGTTATCTTC |
| DB347/VP-AAV/PacI/R | cgccTTAATTAATTACAGATTACGAGTCAGGTATCTGG |
| DB376/VP1(op)/BspEI/F | cggtccggaCCGAAcaaaATGGCAGCAGATGGATACTTACC |
| DB377/VPop/PacI/R | CGGCttaattaaTCACAAGTTTCTGGTTAAGTATCTGGTGC |
| DB348/E2A(op)/SpeI/F | CGCACTAGTaaaATGGCATCCAGAGAAGAGGAAC |
| DB349/E2op/SalI/R | gcgcGTCGACCTAGAAATCGAATGGGTTCTGTCTGG |
| DB307/GFP-ddPCR/F | ACCCTGAAGTTCATCTGCACCAC |
| DB308/GFP/FAM | CTGCCCGTGCCCTGGCCCAC |
| DB309/GFP-ddPCR/R | CCGTAGGTCAGGGTGGTCAC |
| DB406/URA3/dd/F | CAGGATCTGACATTATTATTGTTGGAAGAGGAC |
| DB407/URA3/dd/HEX | CACCCTCTACCTTAGCATCCCTTCCC |
| DB408/URA3/dd/R | GTGCGGTATTTCACACCGCATAGG |
| DB438/Sc-18S/F | GGACGTTTGGTTCTATTTTGTTGGTTTCTAG |
| DB439/Sc-18S/HEX | CTGATGCCCCCGACCGTCCCT |
| DB440/Sc-18S/R | TTCGCAGTAGTTAGTCTTCAATAAATCCAAG |
